# “You can because you do and you do, because you can”: Using interpretative description to examine what it means to be a physically literate adult living with multiple chronic conditions

**DOI:** 10.1371/journal.pone.0297261

**Published:** 2024-01-23

**Authors:** Celeste Petrusevski, MacDermid Joy, Michael Wilson, Julie Richardson

**Affiliations:** 1 School of Rehabilitation Science, McMaster University, Hamilton, ON, Canada; 2 Physical Therapy and Surgery, Western University, London, ON, Canada; 3 Hand and Upper Limb Centre, St. Joseph’s Health Centre, London, ON, Canada; 4 Department of Health Evidence & Impact, McMaster University, Hamilton, ON, Canada; 5 McMaster Health Forum, McMaster University, Hamilton, ON, Canada; 6 Centre for Health Economics and Policy Analysis, McMaster University, Hamilton, ON, Canada; Yarmouk University, JORDAN

## Abstract

**Aims:**

Physical literacy is an emerging strategy to increase participation in movement activities for children and youth, however little is known about how to frame physical literacy for aging adults. The purpose of this qualitative study was to explore how adults with multiple chronic conditions describe physically literacy for adults and to understand the needs, preferences, barriers, and facilitators to acquiring and maintaining physical literacy despite fluctuations in health status.

**Methods:**

Sixteen semi-structured interviews were conducted with working and retired teachers in Ontario, Canada, with varying self-identified physical activity levels and are living with 2 or more chronic conditions. A semi-structured interview guide was used to conduct the interviews. Thematic analysis was used to analyze the data.

**Results:**

Participants identified 5 themes when describing physical literacy for adults: understanding one’s body, conscious commitment to movement, access to and knowledge of rehabilitation health resources, valuable physical activities, and confident problem solver. Results indicate that when acquiring physical literacy for adults, there are important new constructs, such as self-management and the awareness of rehabilitation strategies to maintain mobility, that differ from the traditional physical literacy model.

**Conclusions:**

To improve function and mobility outcomes for adults living with chronic conditions, programs should be guided by a physical literacy framework that addresses the needs unique to aging adults, such as understanding the changes that occur with aging, self-monitoring mobility changes and participating in rehabilitation strategies.

## Introduction

As the global population of older adults increases [1], more people are living longer with chronic health conditions [2]. The prevalence of multiple chronic conditions (MCC), defined as having two or more chronic conditions, continues to increase worldwide, affecting one in three adults [2]. The incidence of MCC markedly increases with age, however over the past decade, the proportion of working-age adults (ages 16–54 years) with MCCs has been steadily increasing [3]. Multiple chronic conditions result in a burden to the patient, including a decline in physical function and mobility [4], and poor quality of life [5]. The increasing prevalence of MCC also creates a significant challenge to the healthcare system, including higher rates of healthcare utilization and medical costs [6].

Lifestyle practices such as decreasing sedentary behaviour and increasing physical activity have demonstrated benefit with the prevention and management of chronic conditions [7–9], however only 52% of Canadians who are 40 to 59 years and 33% of the 60 to 79-years met the physical activity guidelines issued in 2020 [10]. Adults with MCC report many barriers to participation in physical activity, such as cost, time, physical pain and symptoms, lack of guidance from professionals and decreased access to resources [11–13]. Facilitators for participation in physical activity include social interaction, health professional involvement to support physical activity, and health coaching [12].

Rehabilitation providers, such as physical therapists, are experts in restoring function, promoting active lifestyles, coaching, and teaching self-management strategies. However, due to lack of access and affordability for these resources, less than 10% of people who could benefit from rehabilitation services receive them [14]. Increasing access to rehabilitation services is essential to meet the growing needs of our aging population. Innovative approaches are needed, such as rehabilitation self-management programs in functional health using population health strategies to target the mobility and functional needs of aging adults.

Programs designed to improve the physical literacy of aging adults and adults with MCC through health promotion and heath prevention strategies at the individual, community, organizational and service level have the potential to improve important health outcomes for adults and older adults. Physical literacy, most widely defined as “the motivation, confidence, physical competence knowledge and understanding to actively participate in physical activities for life” [15], is an evolving concept, and has been proposed to be a primary determinant of health through its positive influence on engagement in physical activity [16, 17]. Despite inclusiveness being foundational to physical literacy, research in physical literacy has mainly focused on physical education for children and youth and little is known about the benefits of physical literacy for aging adults and adults with chronic conditions [16, 18]. Additionally, the current physical literacy definition is conceptually framed for individuals who are developing and expanding one’s physical activity and does not account for individuals who are experiencing movement and activity constraints due to age- related changes. An integrative review examining which critical components are currently used to frame physical literacy for aging adults (≥45 years) [18], found that physical literacy is defined differently for the older adult population than the current Whitehead definition which focuses on youth and younger adults in the education and sport sectors [16, 18]. Meaningful and/or purposeful activities, knowledge of age-related changes, social interaction and diverse activities were the top four components reported in the literature review when describing physical literacy for adults [18].

Most recently, to further understand what important components should be included in a physical literacy program for adults and adults with chronic conditions, an on-line expert consensus study was completed with key rehabilitation professionals and researchers who were experts in the field of physical literacy [19]. Questions were designed to gain consensus on what components describe a physically literate adult, what rehabilitation principles/strategies should be included in a physical literacy program and how rehabilitation knowledge could be disseminated at a population level to improve function and mobility outcomes for adults. Group consensus resulted in the following top 5 components used to define physical literacy for adults and/or adults with chronic conditions: 1) self-efficacy with movement, 2) confidence in safety of movement, 3) motivation and commitment to physical activity, 4) the ability to self-monitor changes in physical function and 5) understanding the benefits of physical activity and what to do despite physical limitations [18]. This expert consensus study indicates that from the perspectives of healthcare professionals and researchers, re-conceptualizing the current physical literacy definition to include the rehabilitation components required for aging may add value in the promotion of movement and optimal function with aging [18].

To further understand what physical literacy means to adults, research is needed on physical literacy from the perspectives of adults who are living with mobility and physical function challenges. The purpose of this qualitative study was to explore how adults with MCC describe physical literacy for aging adults and to understand the needs, preferences, barriers, and facilitators to acquiring and maintaining physical literacy despite fluctuations in health status.

## Methods

We used a qualitative interpretive description (ID) approach to explore what physical literacy means to adults living with multiple chronic conditions [20]. An interpretive descriptive approach was chosen as the best method as it aims to answer questions of relevance to a clinical discipline in which understanding about the focus of the discipline’s action is considered important. Interpretive description was used to analyze this data because this approach results in a coherent conceptual description that identifies thematic patterns that according to the researchers characterize the phenomenon under study but also reports individual variations within the themes [20]. We wanted to provide an in-depth understanding of the evolving physical literacy concept and to generate results that would enlighten and guide the promotion of physical literacy for adults with multiple chronic conditions [21]. The trustworthiness of the study was assessed by the following: credibility and dependability were addressed by organizing and coding the relevant sections of the interviews into “parent codes”, identifying as initial root codes that occur frequently throughout the data. Transcripts were read again and segments of the content with similar meaning were assigned the same code. The codes were then refined into key themes. Transferability was addressed in the context of the limitations, and confirmability was addressed through member checking and by review of the transcripts and coding by two of the researchers (CP, JR). Ethical approval for this study was obtained from the Hamilton Integrated Research Ethic Board (#8062).

### Sample and recruitment

Following the guidance of interpretive description, purposive sampling was used to interview working and retired teachers. Purposive sampling involves identifying and selecting individuals that are especially knowledgeable about or experiences with a phenomenon of interest. Teachers were identified as the population for this enquiry because they were more likely to have a common understanding of the novel physical literacy construct, through their experience with teaching and curriculum development. Teachers are also likely to have the knowledge and the ability to communicate opinions in an articulate and reflective manner that can help maximize saturation [21].

Participants were recruited between May and November 2021 by advertisement on the Retired Teachers of Ontario (RTO) website, contacting gatekeepers within local community groups (YMCA) and social media advertisement. Participant information was sent via email and interviews were arranged if volunteers met pre-screening eligibility and consent forms were signed. Eligibility for participation in the semi-structured interviews included: 1) adults 40–65 years, 2) currently working or have previously worked as a primary or secondary school teacher, and 3) have been diagnosed with two or more chronic conditions. Adults diagnosed with dementia and individuals who did not speak English were excluded from the study. The sampling strategy involved teachers from 4 different categories; 1) working teachers with moderate to high physical activity (PA), 2) retired teachers with moderate to high PA, 3) working teachers with inactive to low PA, and 4) retired teachers with inactive to low PA, as determined by the International Physical Activity Questionnaire (IPAQ). The sampling strategy allowed a diverse range of teachers to share narrative stories of their experience and what it means to them. The research study is an emergent design and therefore final sample size was determined once saturation of the common themes was reached and no further information could be extracted from the narratives of participants [22]. Before taking part in the study, participants provided informed consent for collection of demographic information and agreed to audio recording of the interview over the Zoom platform.

### Data collection

**Fig 1:**
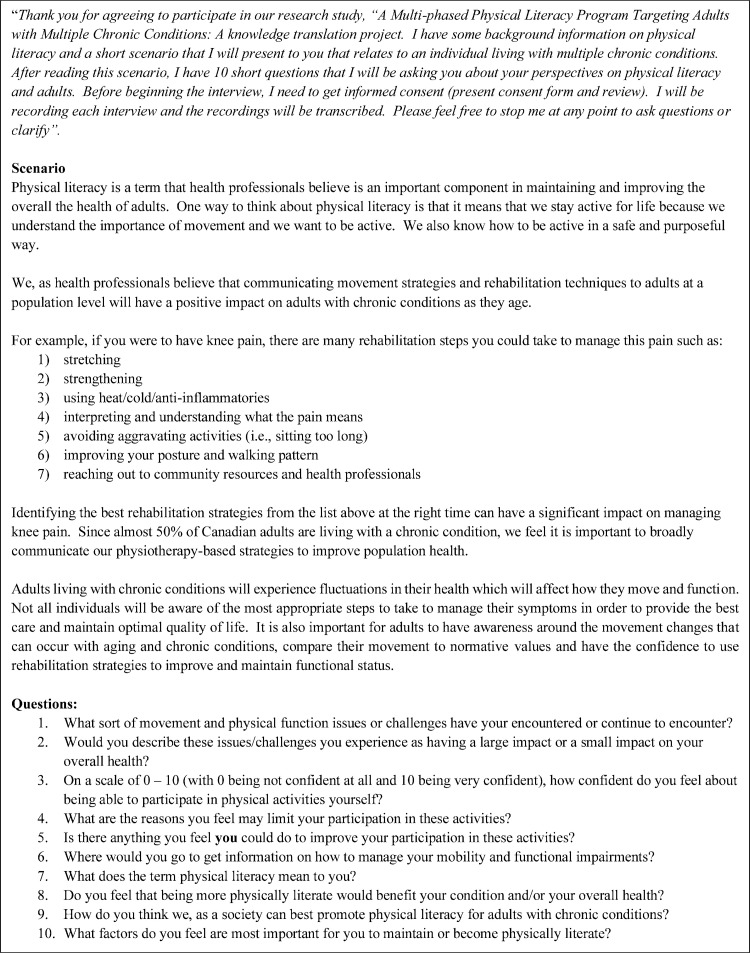
Interview guide.

A semi-structured interview guide was developed by 2 authors (CP and JR) and used to lead the interviews. Following the interpretive description methodology, the interview guide was framed based on the authors rehabilitation clinical experience, recent physical literacy research and the research goals [18, 19]. Participants were read a short scenario, followed by questions examining the following five main topic areas: 1) common mobility and physical function challenges, 2) barriers and facilitators to participation in PA, 3) access to information on chronic conditions, 4) defining physical literacy for adults, and 5) how to share physical literacy knowledge with the public (see Fig 1: Interview Guide). The interview guide ensured that all relevant constructs were discussed, while preserving the necessary openness of qualitative research [23]. Interviews ranged from 50 minutes to 70 minutes and were conducted in November 2021. Only the interviewer (CP) was aware of the participants’ identity during the interview, and all transcripts were de-identified prior to analysis. Participants provided informed consent to take part and have their interview recorded and their quotes used anonymously. Participants were reminded of their right to withdraw from the study at any time.

### Data analysis

All recorded interviews were transcribed using Otter [24], an automatic transcription software for recorded audio/video files. Data was then cleaned (CP), and analyzed using Dedoose, a web-based qualitative content analysis software [25]. Each participant was assigned an ID number. Transcripts were read and re-read by 2 authors (CP and JR) to ensure an understanding of the content within the context of one’s lived experience. A thematic approach that reflected the participants perspective was used to guide data analysis [26]. Relevant sections of the interviews were highlighted and coded into “parent codes”, identified as initial root codes that occur frequently throughout the data. Transcripts were read again and segments of the content with similar meaning were assigned the same code. The codes were then refined into key themes. Coding occurred over 2 months and saturation was reached when no new codes were generated.

To ensure dependability and credibility of analysis initial coding of the first 2 interviews was conducted by author CP and the coding strategy was then reviewed by author JR. This allowed the researchers to discuss any differences and come to a consensus. Authors CP and JR reviewed the codes together in 2 interviews that carefully examined the data behind each code. The remaining interviews were coded by CP who consulted with JR about any uncertainties and resolved through discussion or additional codes emerging from the data. The categorized data were organized around two major headings: a) physical literacy constructs related to adults; and 2) facilitators and barriers to acquiring physical literacy for adults. Differences, and similarities among working and retired teachers, were analyzed by matching the codes with participant descriptors in the Dedoose software.

## Results

### Demographics

A total of 16 interviews were completed individually and virtually using Zoom with 4 participants from each of the 4 sampling groups. Participants in this study were all female (100%) with a mean age of 59 years (ranging from 41–73 years). Fifty percent (n = 8) of the participants were currently working as teachers and the other 8 participants were retired teachers. A summary of participant demographic characteristics is included in Table 1. All participants were diagnosed with 2 or more chronic conditions with arthritis reported as the most common primary condition (50%), followed by chronic neck and/or back pain (17.5%). Five participants (31.3%) lived alone and were either widowed or divorced. Two participants disclosed that they were currently using a mobility aid, due to a recent lower extremity fracture. Four participants (3 retired teachers and 1 working teacher) reported they had joint replacements at the knee or hip in the last 5 years. Thirteen participants reported they had experience with rehabilitation professionals in the past, to address an acute or chronic condition(s). Five main themes emerged from the data related to describing physical literacy for adults: 1) understanding one’s body, 2) conscious commitment to movement, 3) access and knowledge of rehabilitation health resources, 4) valuable physical activities, and 5) confident problem solver.

**Table 1:** Demographic characteristics of interview participants (N = 16).

Characteristic categories	N (%)
**Gender**	
Female	16 (100%)
Male	0 (0%)
**Age**	
40–44	2 (12.5%)
45–49	2 (12.5%)
50–54	2 (12.5%)
55–59	2 (12.5%)
60–64	2 (12.5%)
65–69	1 (6.25%)
70–74	5 (31.25%)
**Work status**	
Working	8 (50%)
Retired	8 (50%)
**Marital Status**	
Married	11 (68.75%)
Single	5 (31.25%)
**Stated primary chronic condition**	
Arthritis	8 (50%)
Cancer	2 (12.5%)
Cardiovascular Disease	1 (6.25%)
Other: Chronic neck/back pain	3 (17.75%)
Fibromyalgia	1 (6.25%)
Neurological condition	1 (6.25%)
**Stated secondary condition**	
Arthritis	6 (37.5%)
Cancer	1 (6.25%)
Cardiovascular disease	1 (6.25%)
Diabetes	1 (6.25%)
COPD	1 (6.25%)
Other: Chronic neck/back pain	4 (25%)
Falls	2 (12.5%)
**Self-evaluated PA level**	
Inactive–Low	8 (50%)
Moderate–high	8 (50%)

### Physical literacy constructs related to aging adults

#### Understanding one’s body

As depicted by Table 2, the theme of understanding one’s body was influenced by four subthemes that centered on having knowledge and awareness around one’s physical and mental health and the changes that occur with aging and chronic conditions. All participants felt that a key component to maintaining or becoming physically literate as an adult is having the knowledge around how physical activity can benefit one’s function and mobility with age and fluctuations in health. Participants explained that it was important to understand which movements may benefit and/or impair their current health condition. Participants explained that having an awareness around the changes that occur with aging and chronic conditions, such as pain, weakness, loss of balance and being able to self-identify these changes and then knowing what steps to take to remediate function and mobility issues was a key component in acquiring physical literacy for adults.

“*I think knowing what my issues are*, *what aches I have and what’s causing them and knowing how to approach them to improve whatever the condition is and getting advice from experts is important*. *I think just being aware of what your body needs*, *and staying on top of things is better*, *as opposed to just saying*, *No*, *I’m too old to do that*. *Make yourself aware of what you need to do to keep going”*. *(ID#1*, *retired 72-year-old woman)*

“*I think*, *being really aware of your conditions and*, *your situation*. *You know*, *maybe you can walk in and do all the things now*, *but you need to understand that if you don’t continue to do that*, *there might come a day where you can’t do it*.*” (ID#6*, *retired 71-year-old woman)*

**Table 2:** Main themes and sub-themes of acquiring physical literacy as an adult.

Themes	Sub-themes
Understanding one’s body	• Staying educated on one’s condition(s) and health status • Self-monitoring functional changes • Reporting/addressing mobility changes • Understanding the impact of PA and nutrition on wellbeing
Conscious commitment to movement	• Active participation in PA along the journey of health • Regulating movement activities with age and illness • Physical health goals • Understanding one’s motivation to move
Access and knowledge of rehabilitation health resources	• Functional health coach • Understanding how to remediate and recover from physical health setbacks • Knowledge of where to go to find physical health information • Public health support
Valuable physical activities	• Social support • Confidence with participating in a variety of safe exercises • Participation in meaningful movements • Environmental awareness with PA
Confident problem solving	• Ability to overcome movement barriers • Ability to try new activities • Ability to adapt to the environment • Resilience with health setbacks

Participants frequently reported that acknowledging one’s physical deficits was a reason for taking up health related activities and movements, despite not participating in activities earlier in life. However, others reported a fear of not being able to keep up with the same activities or causing further injury or pain with participation in exercise and sport.

#### Conscious commitment to movement

Participants overwhelmingly felt that physical literacy for adults has a direct association with an increased commitment to movement. Four sub-themes, including an active participation in physical activity, continually regulating movement activities, setting physical health goals, and understanding one’s motivation to move, guided the main conscious commitment to movement theme. Many participants talked about how “any movement was good movement” and this did not need to involve vigorous physical activity or prescribed exercise. This included staying committed to bettering one’s physical health through setting physical health goals and participating in daily movement activities, even when barriers arise.

“*Being physically literate goes back to what my mom said*, ***you can because you do and you do*, *because you can*.** *This will dictate the quality of my life*. *And*, *you know*, *if I’m not physical and aware of what I can do*, *and I’m not doing it*, *then I’m going to have to accept the consequences*. *And they may not be things I want to accept*, *you know*, *knee pains*, *boredom*, *weight gain*.*” (ID #6*, *retired 72-year-old woman)*

Participants discussed the need to understand individual and environmental moderators for movement, such as personality traits, beliefs about physical activity and socioeconomic factors, such as access to exercise options. Motivation was reported as a critical factor in supporting conscious commitment and participants cited the importance of having awareness about what one’s motivators to move were.

“*I think it is important you understand what your motivation to move is*. *My motivation is to keep moving so that things don’t get worse or actually help some of my chronic issues*. *Also*, *I notice I have better posture when I exercise and that is an extrinsic motivator*. *But my main motivation is the social fun I have when out hiking with the group”*. *(ID #3*, *retired 63-year-old woman)*

As people age, motives that indicate pragmatic or mobility concerns, such as maintaining one’s independence, appear to override motives that are more personally uplifting, such appearance [27].

#### Access and knowledge of rehabilitation health resources

The main theme of access and knowledge of rehabilitation health resources was identified from the following four sub-themes; access to a functional health coach, understanding how to recover from health setbacks, knowledge of where to go to find physical health information, and public health support. The study participants cited that having a trusted expert, or coach in the field of physical activity or rehabilitation was a key component to becoming physically literate as an adult.

“*The most important thing is knowing where you can reach or to whom you can reach for some help when things go wrong*.*” (ID #6*, *retired 71-year-old woman)*

“*I take an anti-inflammatory*, *and that might help*, *and ice and heat*. *I certainly learned in physiotherapy the ice and heat thing*. *So*, *I do that a lot*. *If you do these things every day*, *then it just becomes part of your everyday life*. *It’s just common sense”*. *(ID#4*, *retired 65-year-old woman)*

Many participants talked about their experience with allied health professionals, such as physiotherapists, chiropractors, and massage therapists and how the most important take away from their time with these professionals was the rehabilitation knowledge gained and the increased confidence in having control over their condition. This health coaching appears to support the needed continuation along the physical literacy journey for aging adults.

#### Valuable physical activities

Through qualitative analysis, the main theme, “valuable physical activity” was identified from the following four sub-themes: social support, confidence with participation in a variety of safe exercises, participation in meaningful movements and environmental awareness with physical activity. Participants agreed that a key component to acquiring physical literacy is having an awareness around what meaningful and safe exercises/activities are available for adults who are living with chronic conditions or mobility challenges. Participants reported that they often had to seek out information from friends, family, health professionals and the internet to understand what other adults, with similar conditions are doing to stay active. Even though all participants cited that physical activity was beneficial for aging and chronic conditions, many adults reported that it is important to know what activities are safe for one’s body/condition and to avoid repeating movements that may aggravate pre-existing conditions. Participants explained that it was important to have the confidence to try new activities and challenge oneself in new environments to expand one’s physical abilities. Many participants cited that having an awareness around what exercises and movements bring value and joy to one’s life is an important contributor to building physical literacy as an adult. For example, participants reported it was important to understand if social activities or independent activities bring more enjoyment and create an encouraging environment for facilitating one’s physical literacy.

“*It’s important to recognize what motivates you–for example winning motivates my husband with racquetball–I don’t care about that–I enjoy being social with my friends and how my body feels after I move*.*” (ID #3*, *retired 63-year-old woman)*

#### Confident problem solver

We found that the following four sub-themes were associated with the confident problem solver theme; ability to overcome movement barriers, ability to try new activities, ability to adapt to the environment and building resilience with health setbacks. Participants cited that having confidence to make and sustain feasible changes and overcome barriers, are key factors in becoming a physically literate adult. Given the episodic nature of many chronic conditions, having the self-efficacy to adapt and persevere with movement goals, despite environmental and interpersonal barriers, appears to be integral to building physical literacy for adults.

“*I really liked yoga*, *but after the stroke I didn’t have the balancing abilities*. *So*, *what I had to do is find something that gives me the same results*, *which is flexibility*, *while being safe*. *That’s why I went into Pilates*. *Having said that*, *doing a plank is not a possibility for me because you have to go on your toes*. *So*, *I modify*, *and that’s what I look for in physical activities*. *Like if I can’t do everything*, *I can modify it and still be part of the group”* ***(****ID #6*, *retired 71-year-old woman****)***

Participants commonly expressed their hesitation with pushing beyond their physical comfort zone, due to a fear of re-injury, falling or increasing pain symptoms. However, there was agreement from the majority, that to maintain physical literacy it is important to “own your abilities” and focus on all you “can do” and what strategies you can use to overcome movement barriers.

### Facilitators for acquiring physical literacy

The most frequently cited facilitator for acquiring physical literacy was having social networks and participating in enjoyable activities with friends and family.

“*Yeah*, *I think group programs are really important because you get the social aspect*, *and especially when you’re retired*, *and you live alone*, *you need that social aspect*. *And it kind of motivates you to do more and share information with one another”*. *(ID#1*, *retired 72-year-old woman)*

Other facilitators for physical literacy that emerged included, having access to activities and programs that one enjoys, such as age-appropriate community exercise classes, hiking clubs, pickle ball teams and movement programs that support adults with varying health conditions and abilities. Participants also reported that a strong facilitator for one’s continuing physical literacy journey, despite fluctuations in health, was having credible sources for health information. The McMaster University Optimal Aging Portal was referenced by 4 participants as a trusted online health resource that is actively used to learn about health aging [28]. Participants stated that having a reliable health advocate or coach was important, as they could address changes in health and mobility proactively and start rehabilitation strategies in a timely manner to offset further functional limitations.

“*I’m a nosy type*, *ask questions person*, *you know*, *I want to know about everything about my health*, *but not everybody knows enough to ask the questions*. *People need to be told some of the information without having to ask the questions” (ID#2*, *retired 73-year-old woman)*

Participants reported that previous beliefs from childhood about the importance of exercise were facilitators for physical literacy, however, only 9/16 participants reported being physically active as a child. Participants agreed that acquiring physical literacy could be commenced at any age and only 5/16 participants stated they have been active and physically literate throughout all stages of their life.

### Barriers for acquiring physical literacy

The most frequently reported environmental and social barriers included lack of time, lack of social support and climate changes.

“*I’m basically alone most of time*. *So*, *I’m not going to put myself in a situation that is probably going to get me hurt” (ID# 5*, *retired 64-year-old woman)*

“*I had been prone to pneumonia and bronchitis*, *so I just don’t go out in the cold*. *That’s why I go south*, *so that I don’t have to cope with the winter*.*” (ID#2*, *retired 73-year-old woman)*

One’s beliefs about the consequences of movement or fear of falling was also reported. Even though all participants agreed that physical activity was beneficial for chronic conditions, many adults expressed concern with doing the wrong activity or too much activity, that would then result in previously experienced negative consequences.

“*I’m leery*, *but I want to go skiing downhill*. *But there’s a part of me that’s afraid because if I fall again…*. *The doctor said*, *you’re going to be fine*. *Yeah*, *but it’s still there*. *You know*, *it’s just a reminder from that fall*.*” (ID # 8*, *retired 73-year-old woman)*

Competing health information and lack of health resources was also reported as a barrier to acquiring physical literacy. Some participants stated they received opposing health information from health specialists and on-line health resources, which resulted in a lack of trust and decreased one’s motivation to follow activity guidelines and recommendations. Participants stated they were less likely to participate in movements without access and support from a trusted coach or health professional.

“*The one thing I’m worried about*, *though*, *is not having somebody with me*, *who has the knowledge to correct when I’m doing something incorrectly*, *because I worry about injuring myself more*. *By either overdoing it or not doing it correctly”*. *(ID#1*, *retired 72-year-old woman)*

Participants also reported a lack of community programs available for adults experiencing multiple health conditions. For example, participants reported that they did not “fit in” to the traditional disease specific programs offered (e.g., falls programs, knee osteoarthritis exercise groups), because they were often experiencing competing health issues (e.g., fatigue from cancer treatment) that decreased their confidence in their abilities and the safety of the program. Other participants stated that the disease- specific programs were too focused on the conditions, and they were looking for something more holistic that could challenge them in all areas of mobility and movement.

### Similarities and differences based on working status

Similarities and differences between working teachers (n = 8) and retired teachers (n = 8) were analyzed with the Dedoose software to understand how each represented subgroup described the barriers and facilitators for acquiring physical literacy. When examining the facilitators for acquiring physical literacy, there were similarities between the working teachers and retired teachers, such as having knowledge of the movement options available and how they add value to the management of one’s condition, including continued participation in activities that one enjoys. However, retired teachers reported additional key facilitators, such as having a health coach available to support the journey of physical literacy while navigating multiple chronic conditions and having social support from family or friends. Working teachers reported lack of time and difficulty with navigating multiple conditions as the barriers to acquiring physical literacy. Retired teachers reported barriers such as limited access to physical activity options that met needs and preferences, as well as low confidence with activity and the management of one’s condition. Fear of re-injury, exacerbation of symptoms and fear of falling were commonly reported as barriers to acquiring physical literacy for both working teachers and retired teachers. Refer to Table 3 for a full list of facilitators and barriers for acquiring physical literacy.

**Table 3:** Facilitators and barriers for acquiring physical literacy for working and retired adults.

**Working Teachers**	
Facilitators	• Knowledge of movement options available and how they add value to the management of one’s condition • Participating in activities one enjoys • Knowledge and access to resources help overcome movement barriers • Knowledge of when and where to ask questions
Barriers	• Lack of Time • Navigating diagnosis of new conditions and what is safe • Fear of exacerbating condition
**Retired Teachers**	
Facilitators	• Knowledge of movement options available and how they add value to the management of one’s condition • Health coach available to support PL journey • Social support Participating in activities one enjoys
Barriers	• Unsure where to go to ask questions • Limited physical activity options available that suit needs • Have tried and failed before–low confidence • Fear of falling

## Discussion

This qualitative study had two purposes: first to explore the constructs associated with physical literacy for adults and older adults with chronic conditions and second, to examine the facilitators and barriers for acquiring physical literacy as an aging adult. The semi-structured interview analysis was primarily guided by interpretive description of working teachers and retired teachers’ perceptions of what constructs depict a physically literate adult.

Findings from this qualitative study demonstrated that the constructs associated with physical literacy for aging differ from those associated with the current Whitehead definition of physical literacy [15]. Participants described physical literacy as owning an awareness and understanding of one’s body and obtaining access and knowledge of rehabilitation health resources to acquire the skills needed to confidently problem solve changes in functional health to maintain a conscious commitment to movement and participation in valuable physical activities. Interestingly, common components of the Whitehead definition of physical literacy were identified, such as confidence, knowledge, understanding and engagement in physical activities. However, additional identified key components focused on understanding the changes that occur with one’s functional health, as this relates to aging and chronic conditions, as well as accessing rehabilitation health specialists and coaches to support the physical literacy journey. Lastly, an identified new theme around “problem solving” demonstrates the need to learn skills related to self-efficacy, self-regulation and resiliency with aging that can foster physical literacy with aging, despite health setbacks.

When children and younger adults are acquiring physical literacy, they are continually learning physical competency skills taught from teachers, parents, and sport coaches that match and complement their growing and functionally evolving bodies. However, when adults age and one’s physical functional health becomes more constrained due to age related changes, there is a need for coaches who are experts in the field of rehabilitation and functional adaptation to support and encourage new learning and re-learning of physical literacy skills. Current physical literacy models describe a pathway from birth to childhood where physical literacy skills are developed as a child and then maintained into adulthood. However, this current model may not apply to older adults who are living with age-related or chronic conditions, who likely need support with building new rehabilitation focused physical literacy skills, as opposed to regaining past sports-focused skills. As a promising health promotion strategy to improve function and mobility for aging adults, physical literacy, framed through a rehabilitation lens with the constructs identified in this qualitative study could be a meaningful approach to sustaining positive health behaviours, specifically with adults who are living with multiple chronic conditions. Findings also indicated that an important aspect in promoting physical literacy programing for adults relates to access to quality leaders proficient in motivating adults and older adults to increase their participation in movement activities and self-management and leading the programs within the public health environment.

As in younger populations, confidence related to physical literacy is shaped by past experiences. Therefore, it is important to gain insight into an adult’s physical literacy journey, including understanding which physical activity skills they learned and the context in which they were learned, which skills they may want and need to re-learn, or skills they are confronting for the first time. Previous adverse events such as a fall or fear of exacerbating health conditions during physical activities are barriers that can be mitigated, for example, by learning how to self-monitor one’s balance by practicing activities that challenge stability. By affecting social and cultural norms related to physical activity for people with chronic conditions and overcoming individual level barriers to organized programs and services that support learning new rehabilitation skills that promote participation in lifelong meaningful activity, we will be able to facilitate a deeper understanding of physical literacy for adults.

### Strength and limitations

Strengths of this study include the interpretive descriptive design in which knowledge was co-created by using semi-structured interviews to collect rich data from participants who expanded on their understanding of physical literacy as it relates to their experience with aging and chronic conditions. Additionally, triangulation and member checking ensured the credibility of the study. The transcripts were returned to 4 of the participants for review for feedback about the consistency and validity of the data. However, study was subject to certain limitations. Purposeful recruitment of working adults and retired older adults, from the same profession, teaching, allowed for maximal data saturation for a novel and emerging construct. Teachers have lived experience with the physical literacy construct through their exposure to the school curriculum. Both working and retired teachers were able to thoroughly communicate their ideas in a reflective manner, which added to the value of research. However, this homogenous group of teachers represents a middle to higher socioeconomic group who have access to resources such as private health benefits and therefore are familiar/acquainted with issues such as rehabilitation concepts that many other adults do not. Additionally, the group was comprised of all women, and therefore the views of men were not represented along with limited cultural diversity in the sample.

The small numbers in each group working teachers (n = 8), and retired teachers (n = 8) did not allow for the exploration of sub-group analysis, such as examining different levels of physical activity participation with physical literacy from a more granular perspective. Selection bias may have occurred as a result of purposive sampling by work status. Additionally, those not expressing interest, or those expressing interest who were not interviewed, may have differed from those who were interviewed for the study. The results of the study need to be considered within the limitations discussed and these issues limit the generalizability and transferability of the study; however the results are also likely to resonate with some members of these groups.

### Implications for practice and policy

Publicly funded programs grounded in the emerging physical literacy framework for adults may add value in addressing the mobility and functional health needs of aging adults. Additionally, programs that include middle-aged adults (40–50 years) provide individuals with an opportunity to consider all the changes that occur with aging and initiate preventative plans to address these changes. Community programs should be tailored to improving physical literacy, as defined by the key constructs identified in this study; understanding the role of PA with aging, how to self-monitor mobility, setting functional health goals, learning how to access a health coach, creating confidence with meaningful activities, and understanding how to overcome movement barriers. Improving communication between primary, secondary, and tertiary care as this relates to the role of rehabilitation and physical literacy is needed, along with the continued education of health professionals about promoting physical literacy for aging adults. Lastly, targeting hard to reach populations and individuals who are most in need by providing access to government funded physical literacy programs will make a large impact. Re-branding promotional material from the traditional exercise recommendations to physical literacy material will increase awareness and increase motivation for individuals who may not have been successful with physical activity in the past. Increasing awareness and knowledge of how to be a physically literate adult and older adult through media outlets, and creating collaborations and partnerships with health, education, employment, sport, and public health organizations who share common health goals will help to disseminate the new physical literacy narrative.

## Conclusion

Physical literacy is an emerging strategy to support the management of function and mobility changes associated with aging and chronic conditions. Results from our qualitative interview study with adults who are living with chronic conditions demonstrate that acquiring physical literacy for adults involves the following key components; owning an awareness and understanding of one’s body, obtaining access and knowledge of rehabilitation health resources, acquiring the skills needed to confidently problem solve changes in functional health, maintaining a conscious commitment to movement and participation in valuable physical activities. Further research is needed to understand how integrating the above key components into physical literacy frameworks that support the development of publicly funded programs for adults and adults with chronic conditions will affect the functional and mobility challenges that many aging adults are living with today.
